# Characterisation of Rapid In Situ Forming Gelipin Hydrogel for Future Use in Irregular Deep Cutaneous Wound Healing

**DOI:** 10.3390/polym13183152

**Published:** 2021-09-17

**Authors:** Dewi Utami Nike, Haliza Katas, Nor Fatimah Mohd, Yosuke Hiraoka, Yasuhiko Tabata, Ruszymah Bt Hj Idrus, Mh Busra Fauzi

**Affiliations:** 1 Centre for Tissue Engineering and Regenerative Medicine, Faculty of Medicine, Universiti Kebangsaan Malaysia, Bandar Tun Razak, Kuala Lumpur 56000, Malaysia; nike.dewiutami@gmail.com (D.U.N.); ruszyidrus@gmail.com (R.B.H.I.); 2 Centre for Drug Delivery Technology, Faculty of Pharmacy, Universiti Kebangsaan Malaysia, Kuala Lumpur 56000, Malaysia; haliza.katas@ukm.edu.my; 3 Kumpulan Perubatan Johor Ampang Puteri Specialist Hospital, Ampang, Kuala Lumpur 68000, Malaysia; drfatimahnor@kpjampang.com; 4 Biomaterial Group, R&D Center, Yao City 581-0000, Japan; yo-hiraoka@nitta-gelatin.co.jp; 5 Department of Biomaterials, Sakyo-ku, Kyoto 606-8500, Japan; yasuhiko@infront.kyoto-u.ac.jp

**Keywords:** gelipin, gelatin, genipin, cutaneous wound, injectable hydrogel

## Abstract

The irregular deep chronic wound is a grand challenge to be healed due to multiple factors including slow angiogenesis that causing regenerated tissue failure. The narrow gap of deep wounds could hinder and slow down normal wound healing. Thus, the current study aimed to develop a polymerised genipin-crosslinked gelatin (gelipin) hydrogel (GNP_GH) as a potential biodegradable filler for the abovementioned limitations. Briefly, GNP_GH bioscaffolds have been developed successfully within three-minute polymerisation at room temperature (22–24 °C). The physicochemical and biocompatibility of GNP_GH bioscaffolds were respectively evaluated. Amongst GNP_GH groups, the 0.1%GNP_GH10% displayed the highest injectability (97.3 ± 0.6%). Meanwhile, the 0.5%GNP_GH15% degraded within more than two weeks with optimum swelling capacity (108.83 ± 15.7%) and higher mechanical strength (22.6 ± 3.9 kPa) than non-crosslinked gelatin hydrogel 15% (NC_GH15%). Furthermore, 0.1%GNP_GH15% offered higher porosity (>80%) and lower wettability (48.7 ± 0.3) than NC_GH15%. Surface and cross-section SEM photographs displayed an interconnected porous structure for all GNP_GH groups. The EDX spectra and maps represented no major changes after GNP modification. Moreover, no toxicity effect of GNP_GH against dermal fibroblasts was shown during the biocompatibility test. In conclusion, the abovementioned findings indicated that gelipin has excellent physicochemical properties and acceptable biocompatibility as an acellular rapid treatment for future use in irregular deep cutaneous wounds.

## 1. Introduction

Skin is the most extensive protective layer in the human body that natively hinders the penetration of external pathogens. Any deterioration of anatomical skin structure due to traumatic injuries, burns and abrasion, causes the loss of its integrity and stability. Thus, the external intruders easily penetrate the systemic blood circulation, and the worst-case scenario could cause sepsis and death [1,2]. The current skin wound therapy strategy mainly provides rapid treatment upon injury to promote tissue regeneration while reducing the loss of skin function and preventing chronic wound phenomenon [3,4,5]. As of now, a tissue-engineered skin substitute (TESS) is a gold standard tissue engineering product. It presents less painful procedures and reduces post-operative interventions. Nevertheless, the currently available TESS can not fully resemble native skin due to inadequate angiogenesis and low mechanical integrity. Other limitations, including scar formation, high-end price, long production duration and uneven distribution of pigmentation, have restricted their applications [6,7,8,9]. Therefore, there is a high potential to develop a smart three-dimensional (3D) bioscaffold as an acellular skin substitute to expedite wound closure and tissue regeneration.

Hydrogel has received significant attention due to its ability to absorb wound exudates and provide a moist microenvironment inside the defect site. Its 3D structure offers a suitable environment for cells to attach, proliferate and migrate to support the tissue reconstruction [10,11,12,13,14]. The injectable hydrogel, also has been known as in situ forming hydrogel, is becoming popular since the last decade in the tissue engineering and regenerative medicine field. It can be injected through a syringe or catheter injection in a liquid form and then rapidly polymerised at the injection site. These eminent properties can be applied to a damaged tissue immediately with fewer surgical procedures [15,16,17], which is suitable for complicated and deep irregular wounds.

Various types of biomaterials have been suggested by previous researchers for wound healing applications, for example, natural polymers (gelatin, collagen, hyaluronic acid, etc.) and thermoplastic polymers (polyvinylchloride (PVC), polyhydroxyalkanoates (PHA), etc.). In the current study, gelatin has been selected to develop an injectable hydrogel due to its low price, high availability, high efficiency as well as non-toxic, non-immunogenic, low antigenicity and easily modifiable features. It is generally approved to be used safely by the US Food and Drug Administration (FDA). Abundant amino groups on their molecular chains are essential for cell adhesion by recognising integrin receptors in the cells [15,16,17,18,19,20]. On the other hand, it is mechanically weak due to denaturation and partial degradation at room temperature and dissolves at a temperature above 29 °C [19,20]. Busra et al. [4] mentioned that low mechanical strength affected cellular distribution in 3D scaffolds. In this study, genipin (GNP) was selected as a crosslinker to tailor the properties of gelatin hydrogel due to its excellent mechanical strength, low toxicity, non-immunogenic properties, biocompatible features and ability to extend the biodegradation [21,22,23].

Herein, an in situ experiment forming gelatin hydrogels, that were crosslinked with GNP (Gelipin), have been fabricated. The mix of gelatin and GNP solution will become hydrogel within 3 min at room temperature. The ideal hydrogel to be employed as an acellular skin substitute should be durable, adhesive, able to absorb all exudates, sustain adequate moisture to reduce the risk of scar formation while facilitating epithelialisation and cell migration into the wound, offer mechanical protection and compatible [14,24]. Therefore, the main goals of this study were to evaluate the physicochemical characteristics (injectability, viscosity, mechanical properties, etc.) and biocompatibility (toxicity, viability and proliferation) of gelipin for future clinical applications.

## 2. Materials and Methods

The study design was approved by the Universiti Kebangsaan Malaysia Research Ethics Committee (Code no. FF-2020-017 and FF-2019-504).

### 2.1. Gelatin Hydrogel Formulation and Optimisation

**Scheme 1:**
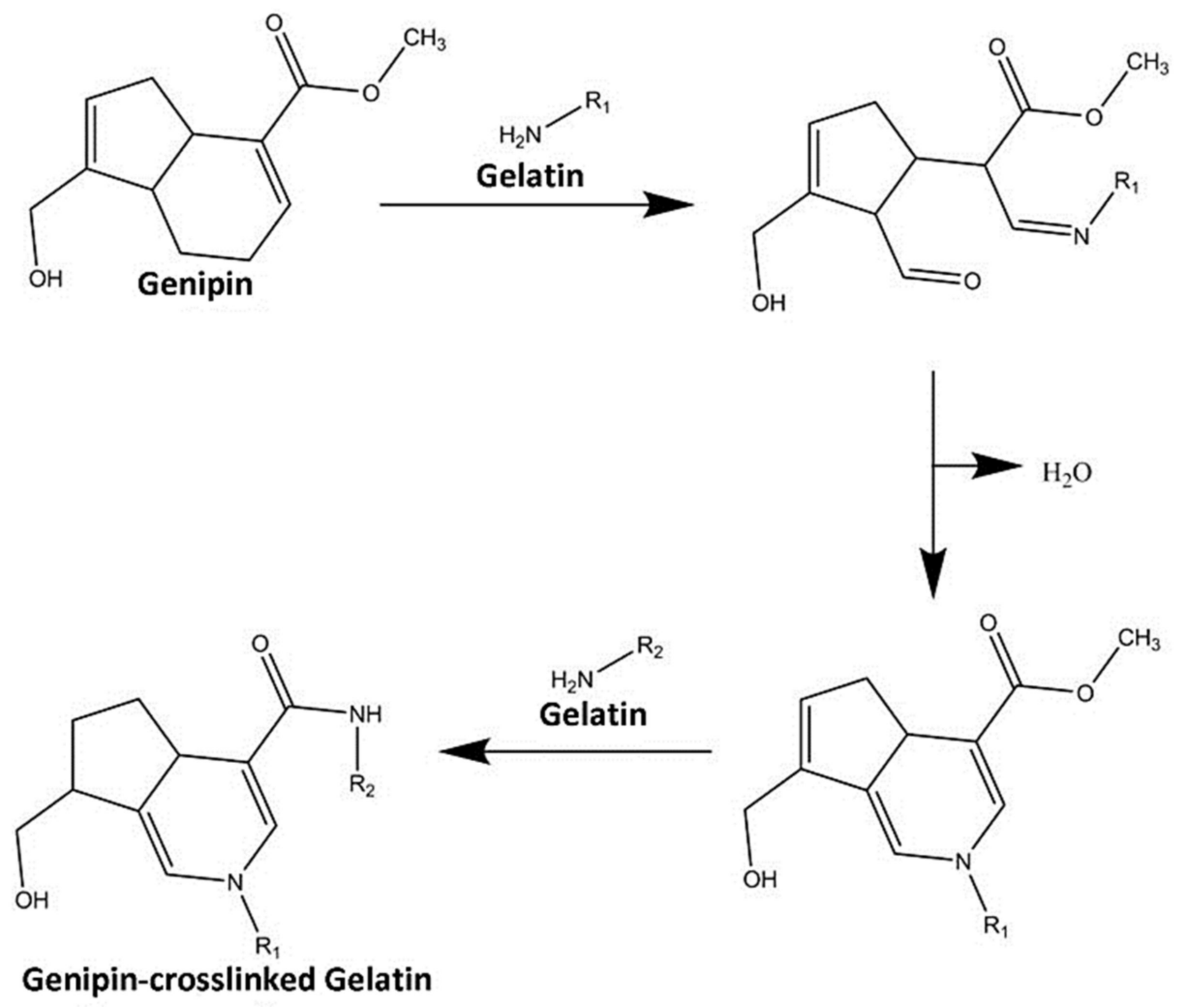
Crosslinking reaction between GNP and gelatin. The initial step is nucleophilic attack of an amine group in the gelatin structure to ester chain in GNP molecule, leading to an open ring of GNP. Another amine group from the gelatin compound then further attacks the methoxy carbonyl group in the GNP structure to produce a crosslinked network.

Gelatin hydrogels (GH) were fabricated, as previously was described by Kirchmajer et al. [25] and Nadzir et al. [26], with some modifications. Three different concentrations of gelatin solution (5%, 10% and 15% *w*/*v*) were prepared by dissolving and stirring gelatin powder (Nitta Gelatin Inc., Japan) in distilled water (dH_2_O) at 40 °C for an hour, 400 rpm by using a hotplate stirrer. Genipin (GNP) solution (3% *w*/*v*) was made by mixing crystallised GNP powder (FUJIFILM Wako Pure Chemical Corporation, Japan) in 70% ethanol (EtOH; MERCK, Darmstadt, Germany) at room temperature (22–24 °C). The GNP solution was added into prepared gelatin solution to obtain three different formulations of GNP-crosslinked GH (GNP_GH) which were 0.1%GNP_GH10%, 0.1%GNP_GH15% and 0.5%GNP_GH15%. The crosslinking reaction is demonstrated below in Scheme 1. Polymerisation time for each formulation was determined via an inverted tube test method at room temperature (22–24 °C) as has been performed elsewhere by Cao et al. [27]. The polymerisation time was recorded through observation from a tilted tube.

### 2.2. Gross Appearance Evaluation

The pictures of the fabricated non-crosslinked GH (NC_GH) and GNP_GH were taken by using a digital camera (Nikon, Tokyo, Japan) immediately after polymerisation.

### 2.3. Fluidity and Injectability

A method, that was established by Sanandiya et al. [28], was implemented with some modifications to investigate the viscosity of NC_GH and GNP_GH. The experiment was performed in triplicate by using a rheometer (Malvern Bohlin Gemini, United Kingdom) in viscometry mode (temperature = 22 °C; measuring gap = 0.5 mm; parallel plate = 20 mm). Gelatin and GNP solution were mixed in 15 mL centrifugal tubes at room temperature (22–24 °C). The mixture was then put at the bottom plate of the rheometer and further evaluated prior to polymerisation. The viscosity value of each formulation was recorded through the integrated software (Bohlin software, GEMINI 200, United Kingdom). An additional step was implied to verify the injectability of fabricated gelatin solution to be successfully passed through the 5 mL syringe as previously was performed by Maulida et al. [29]. The mixture of each formulation was added into the syringe and the initial weight (W_1_) was recorded. Further, the mixture was expelled from the syringe following the recorded weight (W_2_). The following formula was used to calculate the percentage of injectability (I) for each formulation:

% Injectability (I) = (W_2_/W_1_) × 100 (1)

### 2.4. Swelling Ratio

A method, which was established by Thi et al. [30], was applied to analyse the swelling capacity of NC_GH and GNP_GH. This analysis was done to test the ability of hydrogels in absorbing wound exudates. Immediately after polymerisation, the initial weight (W_0_) of hydrogels (n = 3) was recorded and 1 mL of Phosphate Buffer Saline (PBS; 1X, pH 7.4) was added accordingly into the microcentrifuge tube prior to the incubation at 37 °C for 1 h and 24 h. PBS was discarded after the specified time intervals, and the excess buffer was removed slowly by a blotting approach with the usage of filter paper (No. 42, Whatmann^®^, Merck, Darmstadt, Germany). The weight of swollen hydrogel scaffolds (W_s_) were recorded accordingly and the percentage of swelling ratio (S_R_) was calculated using the formula below:

S_R_ (%) = (W_s_/W_0_) × 100 (2)

### 2.5. Biodegradation Profiles

The degradation study of NC_GH and GNP_GH were done by referring to an experiment, that has been performed by Thi et al. [31], with some modifications. These hydrogels (n = 3) were added with 0.0006% collagenase type I (prepared in PBS 1X; phosphate buffer saline) immediately after polymerisation. The initial weight of hydrogels (W_0_) were recorded accordingly and the tubes were placed in the incubator at temperature 37 °C for two days. Every two days, the solution was poured into a waste bottle and the excess solution on the surface of hydrogels was blotted using a filter paper (No. 42, Whatmann^®^, Merck, Darmstadt, Germany). The remaining hydrogel was weighed (W_t_) and the weight loss (%) was calculated by using the following formula:

Weight Loss (%) = [(W_0_ − W_t_)/W_0_] × 100 (3)

### 2.6. Interior 3D-Microarchitectures

Observation of hydrogel microstructures was performed by following some studies, that have been conducted by Treesuppharat et al. [32] and Piao and Chen [33], via field emission scanning electron microscope (FESEM; Zeiss, Supra 55v, Jena, Germany). The lyophilised NC_GH and GNP_GH were coated with an ultra-thin layer of gold/platinum by ion sputtering prior to analysis. The average pore size was measured by using ImageJ software (V1.5, Bethesda, MD, USA).

### 2.7. Porosity

Mun et al. [16] used a solvent replacement method, as previously was optimized, to evaluate the hydrogel porosity. The initial weight (M_1_) of lyophilised NC_GH and GNP_GH were recorded prior to the 99.5% EtOH immersion for 24 h. Then, the excess ethanol was slowly blotted using filter paper (Whatmann^®^, No.42, Merck, Darmstadt, Germany) and the weight of hydrogel (M_2_) was noted. The porosity was calculated by using the equation below:

Porosity (%) = [(M_2_ − M_1_)/(ρ × V)] × 100 (4)

where ρ is the density of 99.5% EtOH and V is the volume of hydrogel.

### 2.8. Moisture Retention

Moisture conservation of NC_GH and GNP_GH were determined as previously has been performed by Chen et al. [34] to observe the capability of hydrogels in retaining moisture environments. The NC_GH and GNP_GH were prepared as previously has been described. The initial weight (W_0_) of hydrogels (n = 3) were recorded prior to the immersion in PBS for 24 h at 37 °C. The excess liquid was then blotted with Whatmann^®^ filter paper (Merck, No.42, Darmstadt, Germany) and hydrogels were placed in a petri dish at room temperature. The swollen weight (W_s_) was recorded after two days. The water retention was calculated using the equation below:

Moisture retention (%) = [(Ws − W_0_)/W_0_] × 100 (5)

### 2.9. Surface Characterisation

To investigate surface properties of NC_GH and GNP_GH, methods that have been established by Loh et al. (2018) [35] was used accordingly. Distilled water was carefully dropped onto the surface of hydrogel and images were captured using a digital camera. The water contact angle was measured by using ImageJ software (National Institute of Health, V1.5, Bethesda, MA, USA) to determine surface wettability. Furthermore, the lyophilised NC_GH and GNP_GHs were subjected to an Atomic Force Microscope (AFM) (Park Systems, NX-10, Korea), Field Emission Scanning Electron Microscope (FESEM) (Zeiss, Supra 55vp, UK) and Energy Dispersive X-ray (EDX) spectrometer (Oxford, UK) for roughness, morphology and elemental contents analysis, respectively. The roughness testing for a 5 × 5 mm sample was performed in non-contact mode scanning with scan rate 0.2 Hz (scan size 5 and 2 nm) and pixel 256 × 256.

### 2.10. Mechanical Properties

The mechanical strength of NC_GH and GNP_GH were evaluated by using a rheometer (Malvern Bohlin Gemini, UK) for oscillation mode in the frequency of 80 Hz and a strain of 0.01% (strain control) at temperature of 22 °C as previously was described by Thi et al. [30]. The prepared mixtures (n = 3) were transferred into the rheometer’s bottom plate, followed by the upper plate (20 mm parallel plate) to lower the measuring gap size of 0.5 mm once polymerised. The value of elastic modulus was recorded accordingly. In addition, the resilience and adhesive force of hydrogel were evaluated by using a texture analyser (Brookfield Engineering Labs Inc., TexturePro CT V1.5, East Bridgewater, MA, USA) in compression mode at a constant speed of 1 mm/s as previously conducted by Chen et al. [34]. The mixtures (n = 3) were prepared in a glass bottle with a diameter of 4 cm and located directly under a texture analyser probe. 

### 2.11. Chemical Characterisation

The functional groups of NC_GH and GNP_GH were assessed by using Fourier transform infrared (FTIR) in the range of 4000 cm^−1^ to 500 cm^−1^ at a resolution of 2 cm^−1^ per point at room temperature (transmission technique). The hydrogels were prepared as has already been explained above and evaluated immediately after polymerisation in a hydrated state. They were placed into a sample holder and immediately scanned. The FTIR spectra were then analysed by identifying each absorbance peak. X-ray diffractometer (Bruker, D8 Advance, Coventry, UK) was addressed to evaluate the crystallinity of NC_GH and GNP_GH with diffraction angle (2θ) in the range of 0° to 60°. The obtained diffractogram was evaluated by using the integrated software (Diffrac. Suite EVA, V4.0, Bruker, Coventry, UK).

### 2.12. Skin Cell Isolation and Culture

Human skin samples were obtained from three patients and further processed as has already been described previously by Busra et al. [4,7]. In brief, 3 cm^2^ skin was minced and cleaned by using sterile Dulbecco’s Phosphate Buffer Saline (DPBS). It was further digested with 0.6% collagenase type I (for 4–6 h) at 37 °C prior to the trypsin-EDTA treatment for 10 min. The cell suspension was then centrifuged for 5 min at 5000 rpm and resuspended with a co-culture medium containing Epilife (Gibco/BRL, Carlsbad, CA, USA) and F12:DMEM (Gibco/BRL, USA) in the same ratio (1:1), which was supplemented with 10% Fetal Bovine Serum (FBS) (Biowest, USA). The cell suspension was then seeded in a six-well polystyrene culture plate and placed at 37 °C in an incubator which was supplied with 5% CO_2_. The medium was changed a week thrice. Human dermal fibroblasts (HDF) were dissociated through differential trypsinisation after the cells reached 70–80% confluency. HDF were expanded in a 75 cm^2^ culture flask with F12:DMEM containing 10% FBS.

### 2.13. Cell Toxicity Assessment

Cytotoxicity test was performed, as has been mentioned elsewhere by Thi et al. [31], towards HDF via LIVE/DEAD cytotoxicity assay for mammalian cells (Thermo Fisher Scientific, Waltham, MA, USA). The hydrogels (n = 3) were fabricated in a 48-well polystyrene culture plate by using sterile gelatin and genipin solution. Immediately after polymerisation, 5 × 10^4^ HDF passage three were seeded on the top of hydrogel prior to the incubation for 24 h. Cell toxicity was examined by using a fluorescence microscope (Nikon A1R-A1, Japan) at 100× magnification after treatment with 500 µL of a mixture of 2 mM acetomethoxy derivate of calcein (calcein-AM) and 4mM ethidium homodimer-1 (EthD-1) at 37 °C for 30 min.

### 2.14. Viability and Proliferation Evaluation

The viability and proliferation of HDF (N = 3) were evaluated by using 3-(4,5-dimethylthiazol-2-yl)-2,5-diphenyltetrazolium bromide (MTT) (Thermo Fisher Scientific, USA) according to the previous experiment that has been performed by Busra et al. [4]. Briefly, 5 × 10^4^ HDF passage three were seeded on the top of hydrogel and MTT reagent was added after 2, 4 and 6 days of incubation prior to the DMSO addition as dissolution reagent. The absorbance was recorded by using a spectrophotometer at 540 nm at specific time intervals.

### 2.15. Statistical Analysis

Graph Pad Prism (V7.0, GraphPad Software Inc., San Diego, CA, USA) was acquired in this research for statistical analysis. One-way ANOVA was employed for multiple group comparison. All values were reported as the mean ± standard deviation. Statistical significance was considered at *p* value < 0.05. All quantitative data values were obtained from triplicate (n = 3) experiments.

## 3. Results

### 3.1. Gross Observation and Injectability Properties

**Figure 1:**
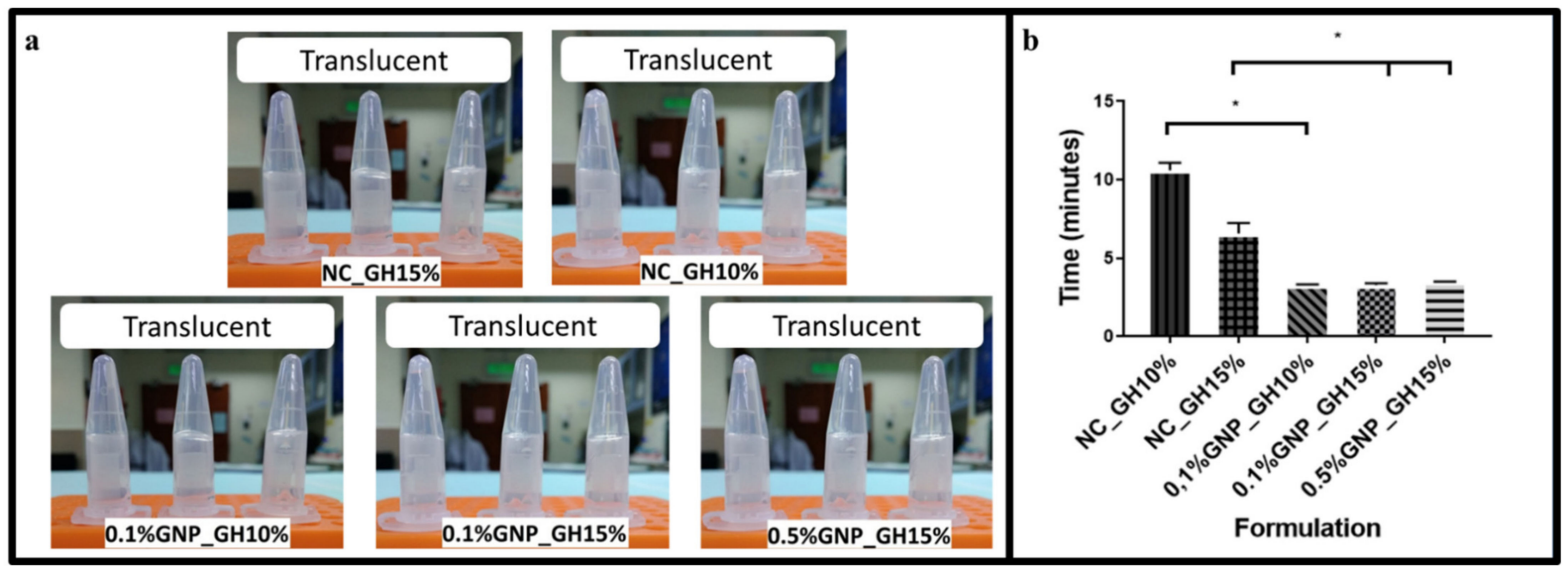
Optimisation phase. (**a**) All hydrogels appeared as a translucent system. (**b**) GNP_GH groups were polymerised within 3 min at room temperature (22–24 °C). * represented significant difference (*p* ˂ 0.05, n = 3, N = 3).

The gross appearance and polymerisation time of non-crosslinked gelatin hydrogel (NC_GH) and genipin-crosslinked gelatin hydrogel (GNP_GH), respectively, were demonstrated in Figure 1a,b. All formulated hydrogels appeared as translucent hydrogel systems at room temperature (22–24 °C). The polymerisation time of GNP_GH was clearly observed within 3 min in 0.1%GNP_GH10%, 0.1%GNP_GH15% and 0.5%GNP_GH15% formulations.

### 3.2. Physical and Biodegradation Properties of Hydrogel

**Figure 2:**
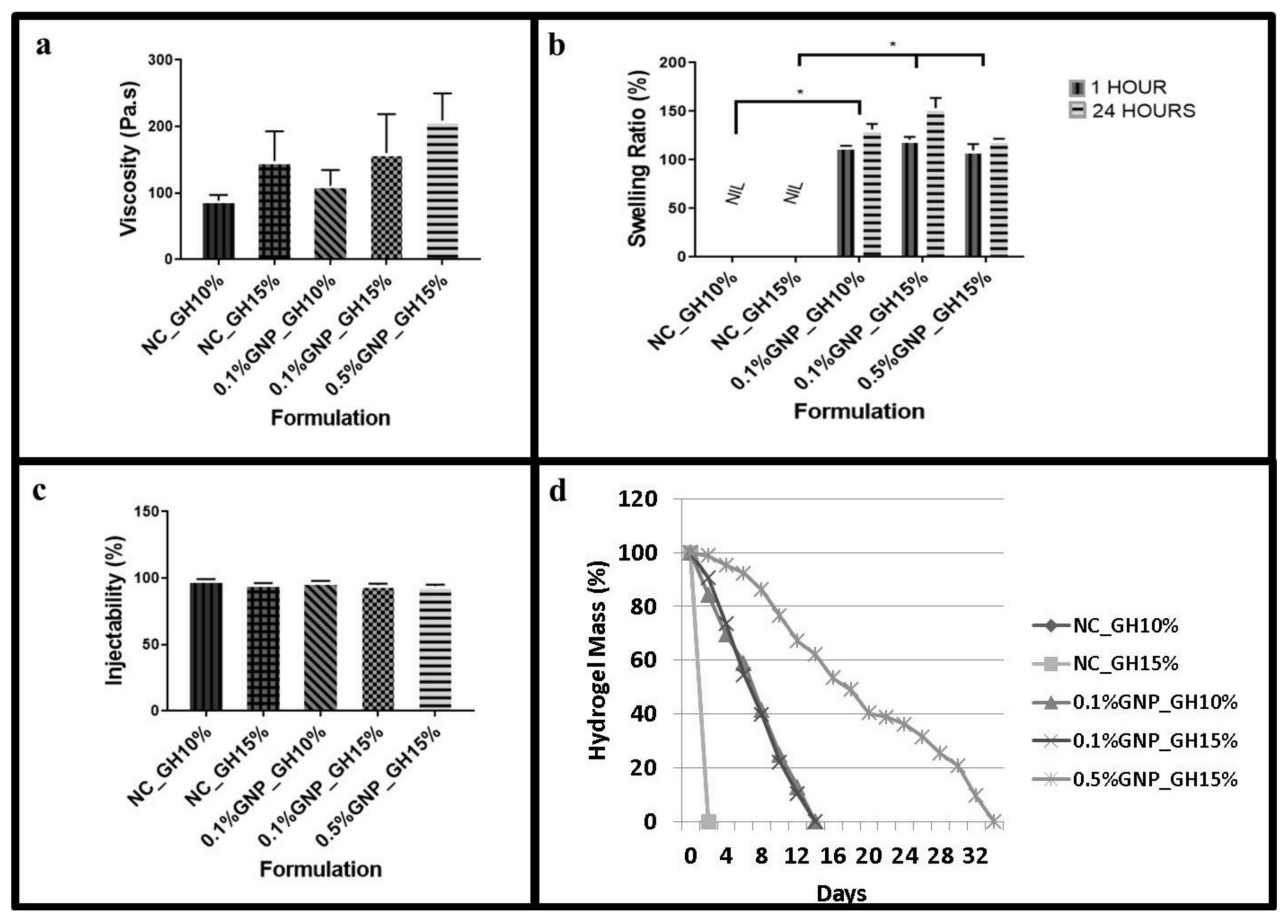
Fluidity, swelling and biodegradation test. (**a**) Viscosity values of GNP_GH were slightly higher than NC_GH. GNP_GH groups were considered as injectable systems. (**b**) NC_GH groups were not stable in PBS solution. Meanwhile, GNP_GH groups exhibited a good swelling ratio (> 100%) after 1 day of PBS exposure. (**c**) GNP_GH groups revealed high injectability (>90%). (**d**) NC_GH groups were not stable in the enzyme environment and GNP_GH groups were resistant against collagenase degradation. * represented significant difference (*p* ˂ 0.05, n = 3, N = 3).

A slight viscosity increment among GNP_GH treatment groups compared to NC_GH control groups, as has been shown in Figure 2a. Even though both of NC_GH10% and NC_GH15% presented a slight change in viscosity, no significant difference in viscosity was recorded. However, the 0.5%GNP_GH 15% (209.17 ± 40.65 Pa∙s) revealed significantly higher viscosity than NC_GH15% (148.1 ± 44.9). The lowest viscosity was demonstrated by 0.1%GNP_GH10% (111.1 ± 23.5 Pa∙s) but no significant difference was identified compared to NC_GH10% (88.73 ± 8.70). The swelling testing revealed that NC_GH control groups were fully disintegrated post-immersion in PBS for 1 h and 24 h. Meanwhile, all GNP_GH formulations exhibited excellent swelling behaviour of more than 100% for both incubation periods as has been presented in Figure 2b. There was a significant difference in swelling capacity between GNP_GH and NC_GH (*p* ˂ 0.05). The 0.5%GNP_GH15% demonstrated the lowest swelling ratio (108.83 ± 15.7%) and 0.1%GNP_GH15% exhibited the highest swelling ratio (121.0 ± 10.57%) after 24 h of incubation. The injectability of GNP_GH groups showed no significant difference compared to NC_GH groups, as shown in Figure 2c. In addition, among GNP_GH groups, the highest and lowest injectability was revealed by 0.1%GNP_GH10% (97.3 ± 0.6 %) and 0.5%GNP_GH15% (94 ± 1%), respectively. According to Figure 2d, all GNP_GH groups were fully degraded minimally within 2 weeks for both 0.1%GNP_GH10% and 0.1%GNP_GH15%, however, only 0.5%GNP_GH15% remained until 34 days of incubation. Meanwhile, both NC_GH groups were completely degraded within 48 h.

### 3.3. 3D-Microporous Structure of Hydrogel

**Figure 3:**
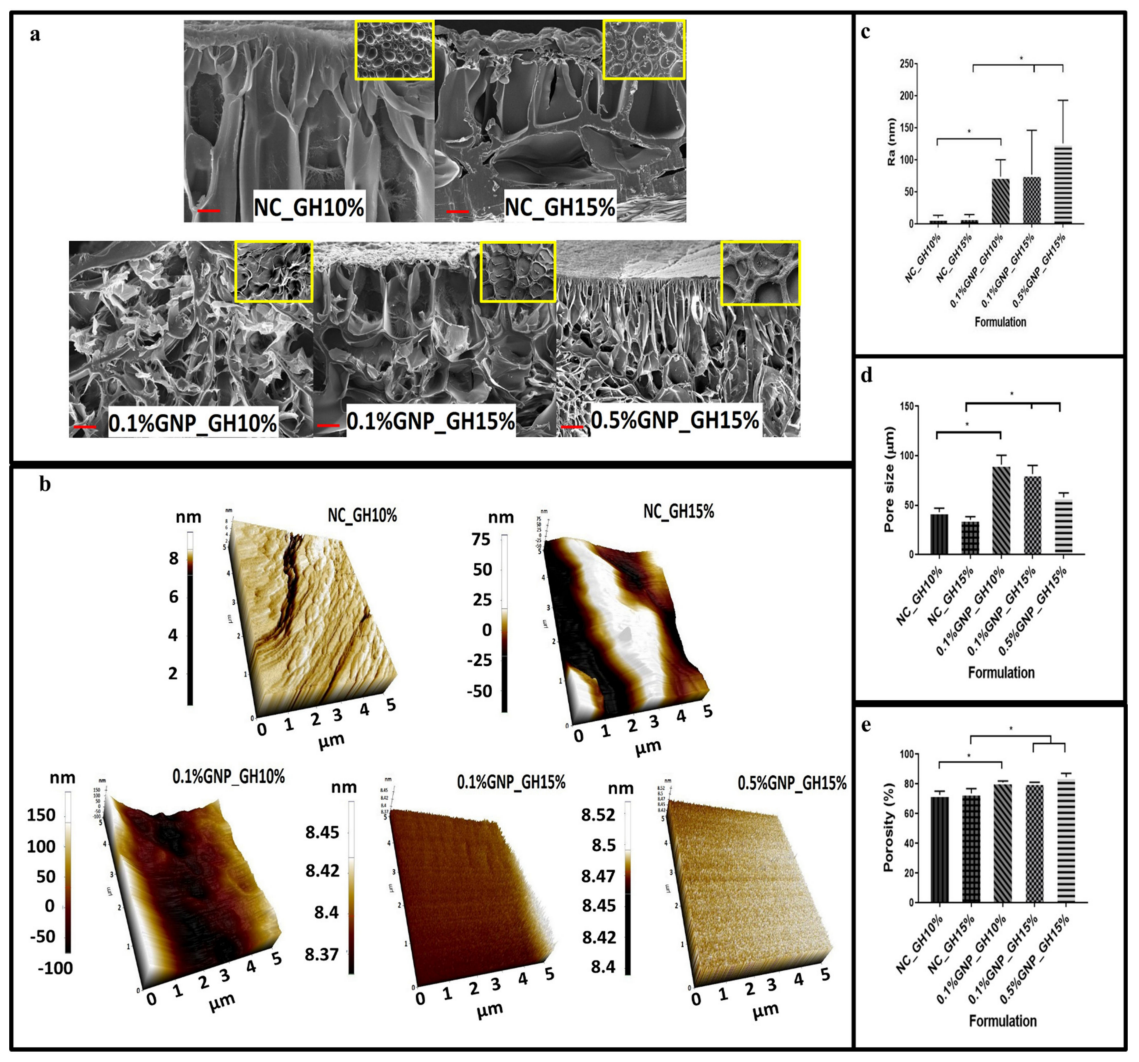
Porous structure, surface morphology and porosity (**a**) FESEM images revealed interconnected porous structures for both the cross section and surface of GNP_GH groups. (**b**,**c**) AFM analysis confirmed the roughness of GNP_GH. (**d**) Pore size of GNP_GH groups were within the range of 50–100 µm. (**e**) Porosity of GNP_GH groups were >70% and significantly higher than NC_GH. * represented significant difference (*p* ˂ 0.05, n = 3, N = 3). Scale bar = 100 µm.

The cross-section and surface images of both NC_GH and GNP_GH groups were illustrated as in Figure 3a. The FESEM photographs displayed interconnected porous structures for all GNP_GH treatment groups. The surface morphology was illustrated as in Figure 3b to represent the surface morphology. In addition, the roughness of 0.5%GNP_GH15% (125.59 ± 67.22 nm) gave higher Ra value than NC_GH15% (10.1 ± 0.9 nm) as shown in Figure 3c and followed by 0.1%GNP_GH15% (76.9 ± 95.9 nm) and 0.1%GNP_GH10% (73.6 ± 32.0 nm). In contrast, both NC_GH groups unraveled the significant lowest roughness compared to GNP_GH groups. Besides, both NC_GH and GNP_GH demonstrated heterogenous pore sizes within the range of 0–100 µm as described in Figure 3d. The pore size arrangement was gradually reduced in size started from 0.1%GNP_GH10% (91.0 ± 9.5) followed by 0.1%GNP_GH15% (81.7 ± 8.5) and 0.5%GNP_GH15% (58.0 ± 4.6). The porosity study revealed that GNP_GH formulations had significantly higher porosity than NC_GH control groups (*p* ˂ 0.05) as shown in Figure 3e. The 0.5%GNP_GH15% and 0.1%GNP_GH10%, revealed the highest porosity (84.67 ± 2.52%) and lowest porosity (80.33 ± 0.58 %), respectively.

### 3.4. Wettability and Biomechanical Characteristics of Hydrogel

**Figure 4:**
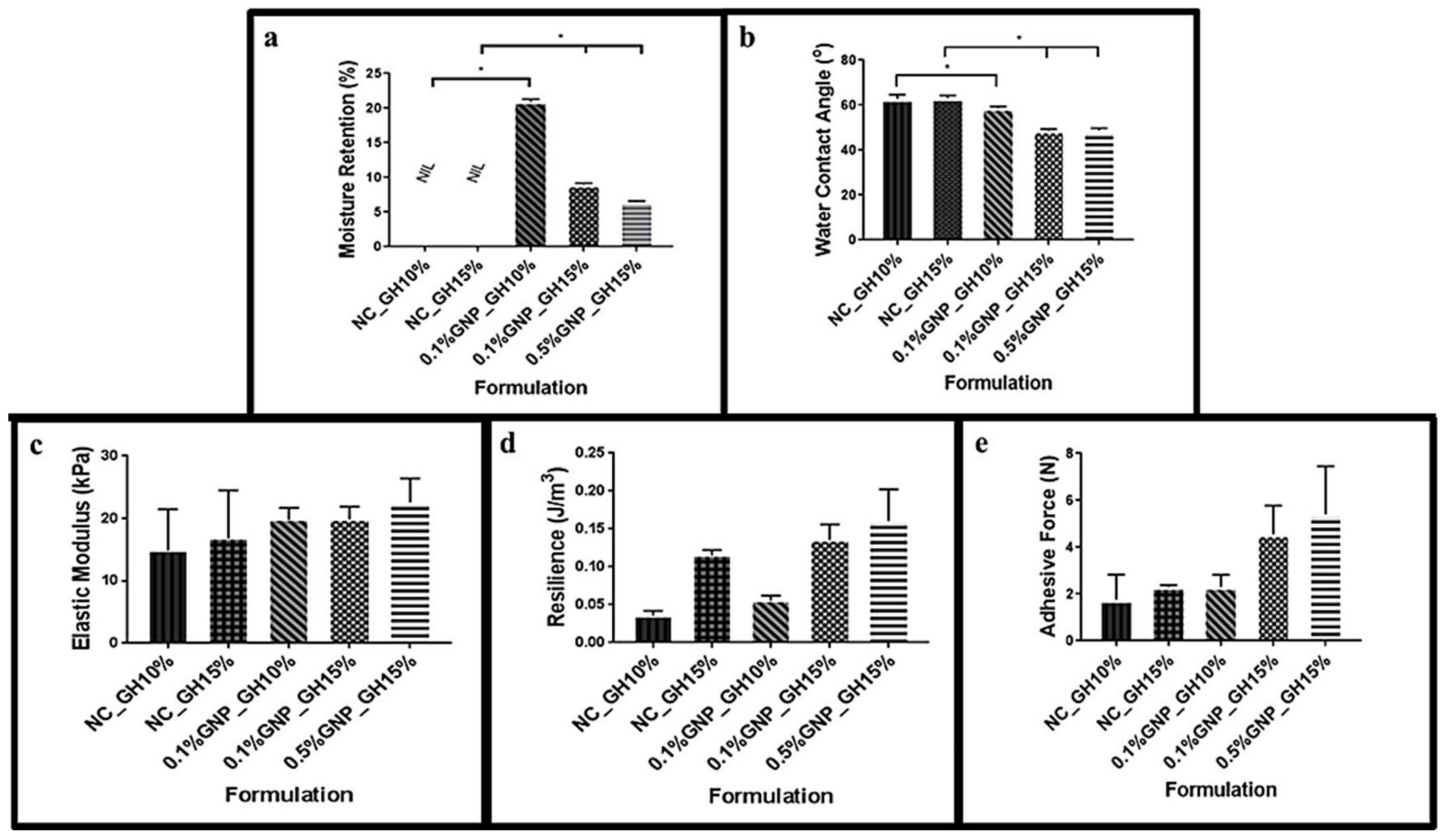
Retention, wettability and mechanical properties. (**a**) GNP_GH was able to conserve moisture and significantly different from NC_GH groups, which were unstable in liquid environments. (**b**) GNP_GH groups were assigned as hydrophilic scaffolds (wettability ˂ 90°) and significantly more hydrophilic than NC_GH groups. (**c**) GNP crosslinking enhanced the elasticity of GH. (**d**) and (**e**) GNP_GH groups were slightly more resilient and adhesive than NC_GH groups. * represented significant difference (*p* ˂ 0.05, n = 3, N = 3).

Figure 4a shown the significant difference in water retention for 0.1%GNP_GH10%, 0.1%GNP_GH15% and 0.5%GNP_GH15% compared to NC_GH control groups (*p* ˂ 0.05) after 2 days of incubation. It can be seen that 0.1%GNP_GH10% (20.9 ± 0.4%) demonstrated the highest retention compared to that of 0.1%GNP_GH15% (9.0 ± 0.20%) and 0.5%GNP_GH15% (6.5 ± 0.2%). The NC_GH were fully dissolved after 24 h. Moreover, all GNP_GH exhibited significantly lower wettability (*p* ˂ 0.05) than NC_GH control groups (Figure 4b). The contact angle revealed the highest value in 0.1%GNP_GH10% followed by 0.1%GNP_GH15% and 0.5%GNP_GH15% were 58.6 ± 0.6°, 48.7 ± 0.3° and 48.5 ± 0.2°, respectively. Besides, the GNP_GH shown higher value in elasticity (Figure 4c), resilience (Figure 4d) and adhesiveness (Figure 4e) than NC_GH control groups, however, there were no significant differences revealed. Finally, the 0.5%GNP_GH15% dominated the elasticitiy (22.6 ± 3.9 KPa), resilience (0.16 ± 0.04 J∙m^−3^) and adhesive within GNP_GH formulations.

### 3.5. Chemical Characterisation of Hydrogel

**Figure 5:**
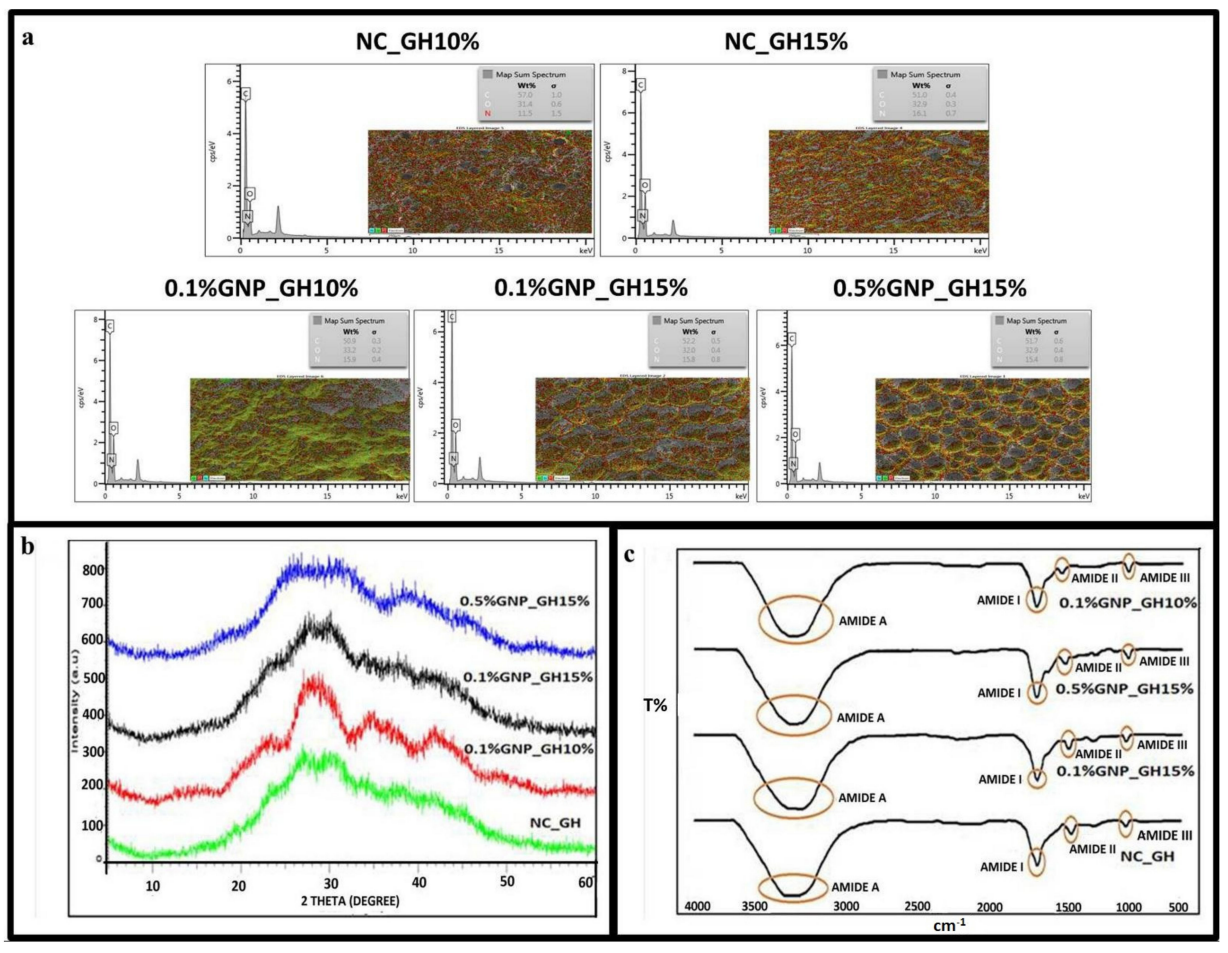
Chemical characterisation. (**a**) EDX map and spectra gave information that the presence of the element in the surface of GNP_GH was similar to NC_GH. (**b**) X-ray diffractogram and (**c**) FTIR spectra confirmed that GNP modification did not alter the origin functional groups and amorphous nature of gelatin.

The elemental study of NC_GH and GNP_GH treatment groups revealed three main components, including nitrogen (N; 11–16%), carbon (C; 51–57%) and oxygen (O; 31–33%), as shown in Figure 5a, represented by blue, red and green colours, respectively. The X-ray diffractogram (Figure 5b) of fabricated hydrogels demonstrated almost similar patterns for both NC_GH and GNP_GH treatment groups. All diffractograms represented a broad peak at 2θ in between 20° to 40° which revealed native gelatin secondary structure. The XRD patterns for NC_GH described NC_GH10% and NC_GH15% due to similar gelatin initial stock except for its concentration. The IR spectra of NC_GH and GNP_GH (Figure 5c) have shown similar absorbances resembling the Amide A (3500–2300 cm^−1^), Amide I (1656–1644 cm^−1^), Amide II (1560–1335 cm^−1^) and Amide III (1240–670 cm^−1^). No major shift was prominent in both XRD and FTIR spectra after GNP modification.

### 3.6. Cellular Compatibility on Hydrogel

**Figure 6:**
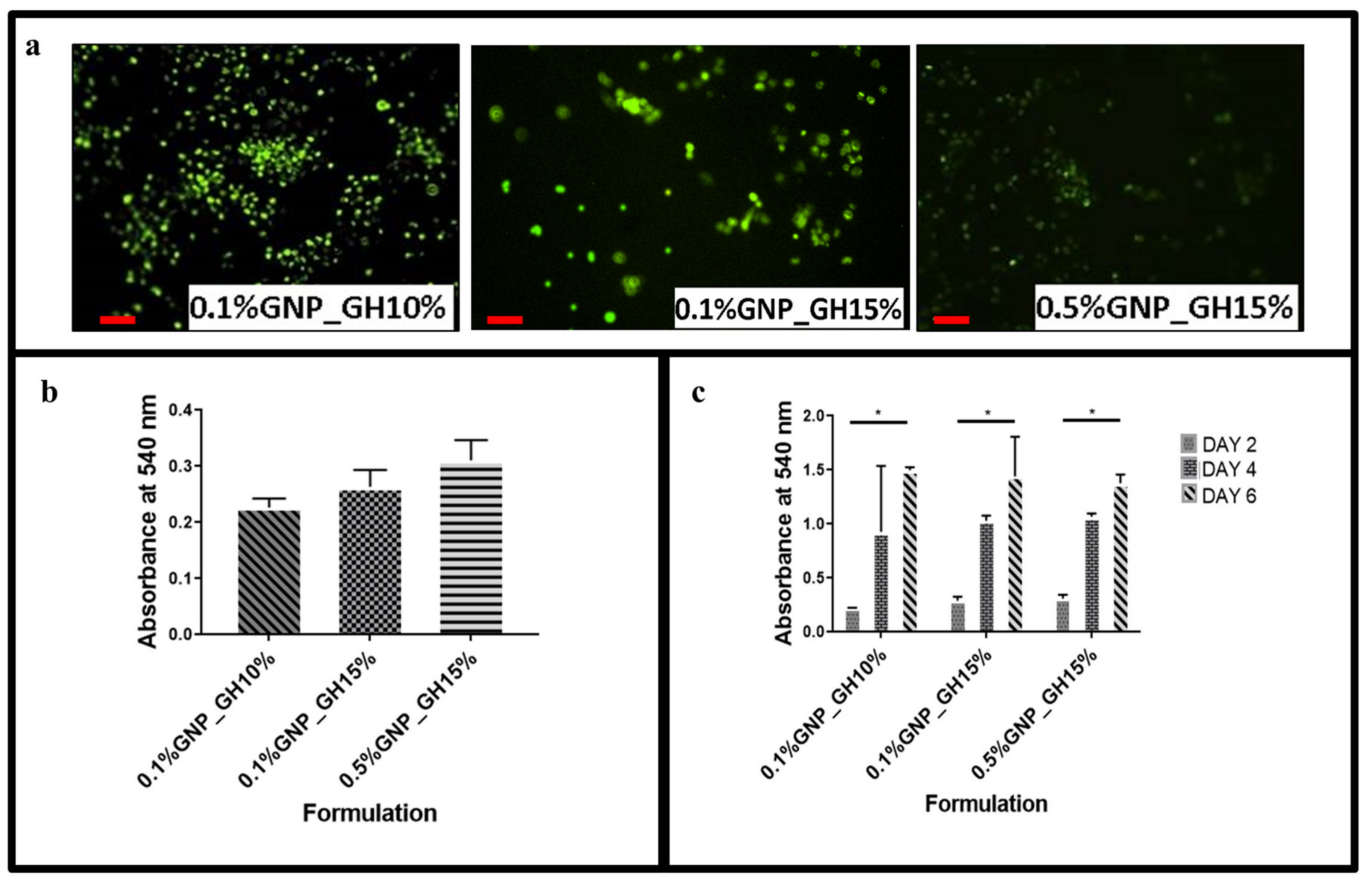
Cellular-hydrogel interaction. (**a**) Live/Dead assay revealed non-cytotoxic effect of GNP_GH. The green color demonstrated live human dermal fibroblasts. (**b**,**c**) MTT assay confirmed that human dermal fibroblasts could proliferate at the top of GNP_GH. * represented significant difference (*p* ˂ 0.05, n = 3, N = 3). Scale bar = 100 µm.

The fluorescent images (Figure 6a) demonstrated that HDF attached to the surface of the hydrogel. The green colour represented viable cells and indicated that GNP_GH was not toxic towards HDF. Figure 6b demonstrated that all GNP_GH groups successfully maintained cell viability throughout 48 h. The highest cell attachment was shown on the top of 0.5%GNP_GH15% which was followed by 0.1%GNP_GH15% and 0.1%GNP_GH10% with no significant difference. Figure 6c revealed that all GNP_GH groups supported the cell proliferation throughout the six days of incubation. The particular data for NC_GH groups were not reported here because their natural shapes were disintegrated in the cell culture environment at 37 °C.

## 4. Discussion

The enhancement of the wound healing process is vital to prevent severe infection and chronic wounds. Thus, the development of a smart bioscaffold for rapid treatment in skin wound application has become critical and challenging [4]. In this study, gelipin—a combination of gelatin (Gel) and genipin (GNP)—was utilised to produce in situ forming gelatin hydrogel (GH). The gel is a cheap biological source with acceptable biodegradation and biocompatibility. Meanwhile, GNP was selected due to its low systemic cytotoxicity and excellent biocompatibility [36,37]. In this experiment, three different ratios of GNP-crosslinked gelatin hydrogel (GNP_GH) (0.1%GNP_GH10%; 0.1%GNP_GH15% and 0.5%GNP_GH15%), which were polymerised within three minutes at room temperature (22–24 °C), were successfully fabricated. The three-minute selection for the polymerisation time was selected in the current study to ensure the clinician/surgeon has enough time to apply them on skin wounds before polymerisation. The polymerisation process occurred due to the helix-coil transition mechanism, which has been reported by Pattinelli et al. [38] and Qiao et al. [39]. In addition, the proposed crosslinking mechanism of action (Scheme 1), that occurred between Gel and GNP, was stipulated and concluded with some modifications from previous articles that were published by Erdagi et al. [40], Wang et al. [41], Muzzarelli et al. [42] and Liu et al. [43], which explored the GNP abilities as a natural crosslinker.

The fabricated hydrogel with low viscosity prior to polymerisation is highly recommended for various wound applications [44]. The viscosity outcome revealed that all GNP_GH groups were within the range of 0.01 to 1 MPa.s, which represented them as flowable material (injected easily). These data were consistent with previous findings that were reported by Rajabi et al. [45] and Zheng et al. [46]. Besides this, GNP_GH groups demonstrated excellent injectability (>90%) [47] to support hydrogel delivery on the defect area before polymerisation. In addition, an injectable hydrogel with high swelling capacity demonstrated a high potential to absorb excessive wound exudates [24,48]. In the current study, all GNP_GH formulations exhibited a high swelling ratio (>100%), which is preferable for skin wound care applications. Through further observation, the use of GNP crosslinking could prevent GNP_GH from enzymatic degradation, as previously has been reported by Ke et al. [49] and Busra et al. [4]. Thus, the GNP crosslinking plays the primary role in slowing down gelatin degradation post-implantation to avoid tissue implantation and reconstruction failure. As has been explored and found by Busra et al. [4], their research concluded that faster degradation would cause the loss of provisional bioscaffolds earlier prior to the production of newly-formed skin tissue. In addition, fully biodegradation post-implantation within 14 days will benefit the tissue regeneration, especially in the dynamic microenvironment in vivo model.

Hydrogel with an interconnected porous structure is preferable to stimulate wound healing phases by facilitating cell migration from native tissue. The optimum pore size for the reconstruction of adult skin should be within the 20–125 µm range [18,50]. The current study demonstrated that GNP and Gel combination has produced an interconnected porous hydrogel with acceptable pore sizes within 50–100 µm. Danilevicius et al. [51] described that an ideal scaffold should have at least 70% porosity for tissue engineering applications to allow sufficient nutrients and cell growth. All GNP_GH groups revealed high porosity (>80%), which is acceptable for skin wound healing and tissue regeneration. Besides this, higher porosity influencing the migration of cells from native towards the implanted area ruling and expedite the wound closure followed by newly-formed tissue.

For skin wound applications, polymerised hydrogel should have appropriate mechanical stability and resemble skin stiffness within the range of 0.06 to 0.86 MPa (6–86 kPa) [52]. In the current study, all GNP_GH groups have shown acceptable elastic modulus values to mimic skin stiffness. Also, it should be highly resilient and offers the best adhesive force. High resilience represents optimum hydrogel elasticity, which is desirable for shape recovery during application to maintain its efficacy [53]. Meanwhile, the higher adhesive force is good for long-term application because the scaffold will integrate longer after being applied to the wound surface Hafezi et al. [54]. Nevertheless, GH is usually featured by low gel strength without any modifications [48,52]. Hence, GNP crosslinking is an essential approach to improve the mechanical strength by creating an intermolecular bridge between gelatin molecules through the covalent bond [24,55]. Thus, they also have demonstrated reasonable resilient and adhesive force properties. The resulted GNP_GH was found to maintain a moisture microenvironment that is essential to accelerate wound healing. Furthermore, it was shown that GNP was responsible for increasing the hydrophilicity of GH. A wettability value less than 90° is good for stimulating skin regeneration [50,56].

The success of bioscaffold development with various materials is primarily can maintain its native properties to avoid any rejection in future clinical settings. Thus, further evaluation was performed to ensure its original structure was primarily still preserved in this study. EDX map and spectra exhibited that no other elements were present upon GNP intervention. FTIR spectra demonstrated four fingerprint peaks of gelatin for both NC_GH and GNP_GH. The absorption vibration between 3500 cm^−1^ and 2300 cm^−1^ referred to amide A, which corresponds to N-H stretching. The amide I region’s band is located at 1656–1644 cm^−1^, which corresponds to the C=O stretching vibration in the amide group coupled to the in-phase bending of the N-H bond the C-N stretching vibration. The amide II region (1560–1335 cm^−1^) corresponds to the N-H bending vibration coupled to stretching C-N vibration. The last region was between 1240–670 cm^−1^ is assigned as amide III (C-O vibration) [57]. An X-ray diffractogram has shown a broad peak for both NC_GH and GNP_GH which indicated their amorphous nature characteristic [58]. Those findings suggested that GNP modification did not significantly alter gelatin’s natural conformation and was consistent with previous results that have been demonstrated by Arif et al. [55].

Cellular compatibility is another concern for an ideal bioscaffold for skin wound treatment to maintain viability and support human skin cells proliferation [50]. Fortunately, all GNP_GH groups indicated a non-cytotoxic effect on human dermal fibroblasts (HDF). They also displayed a positive proliferative effect and were consistent with previous findings from Erdagi et al. [40]. Unfortunately, the proliferation of HDF at the top surface of 0.5%GNP_GH15% was slightly decreased after six incubation days. This phenomenon is probably related to its mechanical strength, as was reported previously by Lee et al. [59]. They concluded that a limited proliferative effect occurred in the hydrogel with higher stiffness due to slower degradation and lower permeability [60]. In addition, the accumulated data of biocompatibility evaluation demonstrated that HDF were able to survive on the rough surface of the fabricated hydrogel.

Table 1 below presents a brief comparison of some hydrogels on the market for wound healing with gelipin [60]. It can be concluded that gelipin provides better features for irregular deep wounds.

## 5. Techno-Economic Challenges

Some challenges are experienced during the research and development process of any medical device development prior to commercialisation. The production of gelipin has its own potential pros and cons which are started from the initial stage, safety evaluation, efficiency via preclinical model prior to clinical trial near future. In addition, medical devices are always haunted by the high cost of production and expensive selling price due to the high quality of clinical-grade products. Their production process requires strict rules and regulations with high-end facilities. However, gelipin was designed to equip clinicians for treating particular patients suffering from conditions such as traumatic injury or chronic wound including diabetic ulcer with lack of tedious preparation, one-time application to defect area and lesser rejection rate. The frequent changes of any such products, which is compared to one-time application product, may contribute to high biological hazard production and also accumulates frequent visits to the hospital or clinic for wound management that are costly and cause public healthcare burdens. Our proposed gelipin product comprises of the main natural components: gelatin (mammalians) and genipin (natural flower). The gelatin was manufactured from green resources; animal waste-product from slaughter-house that could reduce the severe pollution jeorpardising current green environment stability. A future direction for gelipin improvement may be looking into the innovation of gelipin preparation by using the microwave approach without impairing its native structure by using a manufacturing kit in powder form for long-term stability in product quality and reduced cost in terms of commercialisation purposes.

## 6. Conclusions

In conclusion, in situ forming gelipin, which was polymerised within three minutes at room temperature (22–24 °C) resembling future clinical application, has been successfully developed. The gelipin (GNP_GH) was proven to be injectable, tough, resilient, adhesive, hydrophilic and durable through physicochemical characterisation. Furthermore, it provided high swelling capacity, moisture retention capability and interconnected porous structure to support the wound exudate absorption. Lastly, it was found to be non-toxic and biocompatible towards human dermal fibroblasts (HDF). The abovementioned properties are desirable in pharmaceutical and clinical applications. Therefore, in situ forming gelipin hydrogel is a promising candidate for a rapid acellular treatment on-the-shelf product and potentially containing any additional drug/biomolecule/growth factors to expedite the wound healing process.

## Figures and Tables

**Table 1:** Comparison of gelipin with commercially available hydrogels.

	Gelipin	Purilon^®^ Gel (Coloplas Ltd.)	Derma-Gel^®^ (Medline Ind. Inc.)	Intrasite^®^ Gel (Smith & Nephew)
Precise filing for deep wound	✓✓	✓	✓	✓
Durable	✓✓ (14 days)	✓ (3 days)	✓ (5 days)	✓ (3 days)
Adhesive	✓✓	✓	✓	✓

## Data Availability

The data presented in this study are available on request from the corresponding author.
